# The Musical Structure of Time in the Brain: Repetition, Rhythm, and Harmony in fMRI During Rest and Passive Movie Viewing

**DOI:** 10.3389/fncom.2019.00098

**Published:** 2020-01-21

**Authors:** Dan Lloyd

**Affiliations:** Department of Philosophy and Program in Neuroscience, Trinity College, Hartford, CT, United States

**Keywords:** fMRI, graph theory, time, temporality, oscillation, rhythm, harmony, music

## Abstract

Space generally overshadows time in the construction of theories in cognitive neuroscience. In this paper, we pivot from the spatial axes to the temporal, analyzing fMRI image series to reveal structures in time rather than space. To determine affinities among global brain patterns at different times, core concepts in network analysis (derived from graph theory) were applied temporally, as relations among brain images at every time point during an fMRI scanning epoch. To explore the temporal structures observed through this adaptation of network analysis, data from 180 subjects in the Human Connectome Project were examined, during two experimental conditions: passive movie viewing and rest. The temporal brain, like the spatial brain, exhibits a modular structure, where “modules” are intermittent (distributed in time). These temporal entities are here referred to as themes. Short sequences of themes – motifs – were studied in sequences from 4 to 11 s in length. Many motifs repeated at constant intervals, and are therefore rhythmic; rhythms, converted to frequencies, were often harmonic. We speculate that the structure and interaction of these global oscillations underwrites the capacity to experience and navigate a world which is both recognizably stable and noticeably changing at every moment – a temporal world. In its temporal structure, this brain-constituted world resembles music.

## Introduction

In science, “ontology” denotes the determination of the relevant categories and objects available to observation and hypothesis formation (Chakravartty, 2017). Historically, scientific ontologies have divided space first, and only later, time. This is vivid in neuroscience: From the Latin-fluent anatomists to Brodmann to the physical Connectome (Sporns et al., 2005; Hagmann et al., 2007), neuroscience continues to deploy a rich spatial ontology. Temporal ontologies in neuroscience are comparatively recent, but equally rich. An exemplary building block of temporal ontology rests on the exploration of oscillatory neural signals. Fourier analysis affords a powerful descriptive vocabulary which has been abundantly employed in neuroscience. (The references are too many to list in one place, but will appear throughout this paper). The Fourier Transform (FT) has moreover inspired a family of wavelet transforms (WT). These techniques have been abundantly exploited in the study of the temporal brain (see section “Oscillation, Information Broadcasting, and Maintenance” for discussion). However, does the FT/WT framework provide a complete temporal ontology for the brain? Are there other temporal structures, beyond the FT/WT package, that are detectable in brain image series? Here, we will borrow a few basic concepts from a domain where time is centrally important: music. Many musical concepts are essentially temporal, involving order, duration, and temporal relationships in their definition. Centuries of music theory and musicology (including cognitive musicology) afford reasonable criteria for their measurement (Sethares, 1998, 2007; Lloyd, 2011; Huron, 2014). Might they or their analogs apply to neuroimaging time series?

New departures in method necessarily involve exploratory data analysis, and new ontologies particularly involve starting afresh. There are therefore many possible directions for this paper to take (Hutchison et al., 2013; Allen et al., 2014; Calhoun et al., 2014; Kopell et al., 2014). Some plausible starting points and assumptions are highly negotiable. Their motivation will be reviewed in the methods section, but are also discussed in sections “What now? What’s next?”; “Oscillation, Information Broadcasting and Maintenance”, “Rhythm”; and “Harmony”. Here, we first develop a “temporal parcellation” of the imaging data to be examined. That is, we extract a preliminary differentiation of the temporal landscape, just as spatially rooted dynamics rests on a spatial parcellation as its foundation. Graph theory is one method (among many) that can be applied here, but with a pivot from space to time. In its spatial (standard) application, graph theory begins with a set of spatially discrete entities, called nodes (brain regions, usually), and some measure of linkage (edges) between them (often correlation) (Sporns et al., 2005; Hagmann et al., 2007; Bullmore and Sporns, 2009; Sporns, 2011a, b; cf. Stanley et al., 2013; Fallani et al., 2014). The links are thresholded in order to define adjacency among the nodes. Working from the adjacency graph, various communities of nodes can be distinguished, along with other network properties of interest. These go by different names: modules, networks, communities, clubs, or cliques, among others. They can be defined in various ways, but one common measure of modularity discovers groups that have many interconnections among the nodes within the group, but only sparse connections between the groups (Bullmore and Sporns, 2009; Rubinov and Sporns, 2010; Sporns, 2011a, 2012).

Network analyses have usually been employed spatially or spatiotemporally. Spatial network analyses begin with spatially delineated regions (nodes). The time series of activation at all nodes are correlated over the entire time course of an experimental condition for one or more subjects, forming the basis of the resulting graph or network. Spatiotemporal modularity posits that the functional relationships among nodes are variable over time. For example, the same correlational measure might be applied along a sliding temporal window to generate a sequence of modular parcellations, a dynamic functional connectome (Hutchison et al., 2013; Allen et al., 2014; Calhoun et al., 2014). This temporal sensitivity nonetheless rests on an initial spatial parcellation or a sequence of spatial parcellations.

In contrast to both these applications, in this study the graph-theoretic analysis is exclusively based on temporal features in data. Or in other words, there is no initial spatial segregation; the region of interest is simply the brain in its entirety, and the similarity measures are applied exclusively along the spatial dimensions. Instead of regional/spatial nodes, the foundational entities are temporal, namely, individual whole brain images, captured via fMRI, at each moment in time in the series of images. These fully temporal “nodes” might well be called “moments.”

**FIGURE 1:**
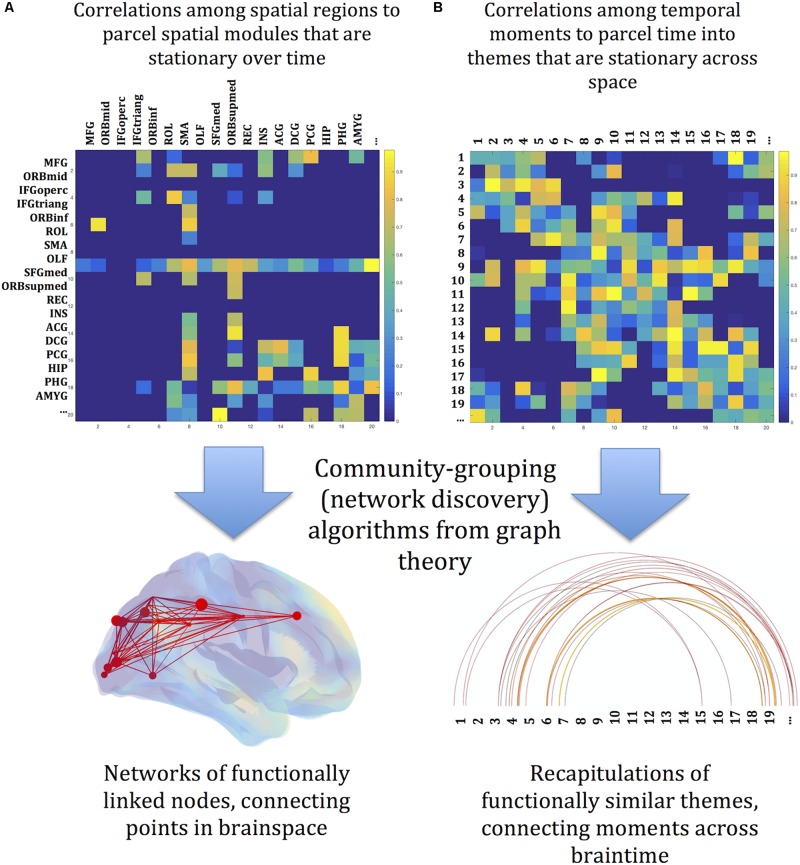
Conceptual sketch of the reorientation of graph theory from space to time. **(A)** Graph theory is a powerful tool for discovering networks among spatially localized modules. Their starting point is an association matrix relating identified regions of the brain to each other, usually through measures of correlation of activity over time. Network discovery algorithms find the spatial network links among the identified regions – the spatial connectome. **(B)** The same tools are here deployed across time. The starting point is an association matrix, but here the associations are between identified moments in time. Network discovery algorithms link sequences of images into themes, which recur as the experiment unfolds. Recurrence and recapitulation are the temporal analogs of spatial network connections. The profile of linked themes is a map along the dimension of time – the temporal connectome. The brainy depiction of the spatial connectome and the linear depiction of the temporal connectome are arbitrary conventions for visualizing quantitative relationships among states of brain activity.

Despite the application of graph theory, the complete pivot toward time translates the spatial concepts inherent in graph theory as spatial metaphors for relations in time. Adjacency among moments is measured by their spatial correlation (across all the voxels of each image, compared image to image), rather than temporal correlation of time series recorded at spatially distinct sites. By this measure moments that are separated by long intervals might nonetheless be adjacent. The equivalent of a module, then, might be distributed in time, and such modules might interweave; the spatial connotations of the term “module” is misleading in this context. We propose instead to refer to these collections of correlated moments as themes. One theme might be present for a single uninterrupted interval, or it may be distributed temporally among other themes. In short, a theme comprises timepoints where patterns of global brain activity are similar, and divisions among themes are determined by the modularity algorithm. The sequence of thematic instantiations comprises an overall thematic profile of an image series. Subsets of the overall thematic profile, short continuous sequences of thematic instantiations, are motifs. [Note that this usage differs from “motif” in network analysis (Milo et al., 2002)] The strategy for this analysis is sketched in Figure 1, with a division between a space-first parcellation (A) and a temporal parcellation (B).

This complete pivot toward time foregrounds the structure of time in the brain, without assumptions about spatial structure. The first and fundamental question, then, is simply: Is there temporal structure in fMR image series? This will offer a data-driven ontology of temporality in the brain. It will be useful, however, only if we can meaningfully describe observed temporal organization. There are many paths to follow, some of them to be discussed in specific contrasts with the methods here; the path in this paper, toward quasi-musical properties, does not exclude other approaches. If there is an anatomy of time, this global temporal parcellation can then provide a data-driven clue to the spatial divisions most relevant to the dynamic functional connectome (Zuo et al., 2010).

## Materials and Methods

For this analysis, data from 180 adult subjects (ages 20–35, 108 Female) in two scanning conditions were downloaded from the Human Connectome Project 1200 Subjects Data Release, May 2018 (HCP^1^; data repository^2^; Marcus et al., 2011; Van Essen et al., 2013; Hodge et al., 2016). Subjects were scanned on a Siemens MAGNETOM 7T MR scanner housed at the Center for Magnetic Resonance (CMRR) at the University of Minnesota in Minneapolis, MN, using a Nova32 32-channel Siemens receiver head coil. Whole-brain sequence gradient-echo EPI images were acquired with the following parameters: TR 1000 ms; TE 22.2 ms; flip angle 45 degrees; FOV 208 × 208 mm (RO × PE); Matrix 130 × 130 (RO × PE); Slice thickness 1.6 mm; 85 slices; 1.6 mm isotropic voxels; Multiband factor 5; Image Acceleration factor(iPAT) 2; Partial Fourier (pF)sampling 7/8; Echo spacing 0.64 ms; BW 1924 Hz/Px^3^.

The two conditions studied here are: (1) resting with eyes open, 900 images (15 min), using the first of four imaging sessions with each subject, and (2) passive movie viewing, 900 images, using the first of four imaging sessions in that condition (Smith et al., 2013). The audio/visual movie was a compilation of short excerpts from Vimeo videos available under Creative Commons licensing^3^.

### Preprocessing

Images were preprocessed using HCP minimal preprocessing pipelines (Glasser et al., 2013; Marcus et al., 2013). This includes three structural pipelines: PreFreeSurfer, to create an aligned and undistorted structural volume space for each subject and register subjects’ spaces to MNI space; FreeSurfer, to parcel volumes into predefined structures, reconstruct white and pial cortical surfaces, and register surfaces to FreeSurfer’s surface atlas, fsaverage; PostFreeSurfer, performing individual surface registration using multimodal surface matching (MSM), based on areal features including sulcal depth, myelin, and functional connectivity maps.

Then, the fMRI Surface pipeline mapped the cortical gray matter voxels onto cortical surface vertices, and subcortical volume voxels, to standardize the surface and subcortical “grayordinate” space for all subjects. [Among other advantages, mapping via surface vertices greatly reduces the number of datapoints needed to express 7T images, making further computational analysis feasible (Glasser et al., 2013). These data were smoothed with a surface algorithm to 2 mm FWHM (Glasser et al., 2013). Finally, 7T rfMRI 4D volume and grayordinate (surface vertices + subcortical voxels) Data, and 7T movie data, were further preprocessed to remove structured artifacts using FSL’s FIX (FMRIB’s ICA-based Xnoiseifier, Salimi-Khorshidi et al., 2014). For details, see Glasser et al. (2013, 2016) and the HCP 1200 Subjects Data Release Reference Manual^3^. The preprocessed data sets were downloaded from HCP during May 2018.

### Graphs

Association matrices were constructed using partial correlations between all voxels in each image series, while controlling for their mean activation (Marrelec et al., 2007; Smith et al., 2011; Hutchison et al., 2013; Varoquaux and Craddock, 2013; Epskamp and Fried, 2018). Each matrix was converted to binary (undirected) connection graph by thresholding the matrix to preserve the top 5% of inter-node values (Rubinov and Sporns, 2010). These binary association matrices were the basis for all subsequent analyses. In traditional network analysis, these steps would be applied to spatially distinct regions (usually anatomically defined regions of interest). Here, the entities to be linked are not spatial but temporal. Instead of measuring correlations of time series between regions, we measure correlations of spatial patterns between time points. The overall analysis is sketched in Figure 1, with a division between a space-first parcellation (A) and a temporal parcellation (B). Each whole-brain image is a node (a “moment”) in a temporally connected network, to be analyzed with graph theoretic methods. All the measures described herein were assessed for significance by contrast with baseline random images that preserve the degree distribution of the original data (“null-hypothesis networks,” Rubinov and Sporns, 2010). That is, the distribution of the numbers of links originating from nodes remains constant while the pairings of the connection matrix are varied randomly (Maslov and Sneppen, 2002). The randomizing function and other functions are found in the Brain Connectivity Toolbox (BCT^4^ Rubinov and Sporns, 2010). In all cases except where noted 100 baseline association matrices were generated for each subject to be contrasted, subject by subject, with the actual data. Appropriate corrections for multiple comparisons were applied using the Bonferroni method.

### Theme and Modularity

Modularity in network analysis denotes the subdivision of network nodes into non-overlapping groups where similarity is greatest within the group, and minimized between the groups (Rubinov and Sporns, 2010). The temporal analog of the module is the theme, which comprises moments of similarity among the full brain images, as assessed through the association matrix described above. A temporal theme is conceptually quite different from a spatial module; nonetheless various measures of modularity can be applied. Modularity was measured using two standard measures (Girvan and Newman, 2002; Newman, 2006; Reichardt and Bornholdt, 2006; Blondel et al., 2008), also implemented through BCT. Modularity assessments with these two algorithms were very similar, and so further analysis was based in the Louvain group method of Blondel et al. (2008). In addition to providing the optimal group divisions, the modularity algorithms calculate a statistic quantifying the degree to which the network can be subdivided into groups with high ingroup similarity and low outgroup similarity (Kaiser, 2011). We compare this statistic for each subject with the same statistic generated for 18,000 surrogate association matrices (100 per subject). Similar analyses, including baseline contrasts, were conducted for both the rest and movie conditions.

This method is conceptually similar to several recent proposals for deriving network architecture from time series data (Voss et al., 2004; Lacasa et al., 2008; Shirazi et al., 2009). Lacasa et al. (2008) describe a “visibility graph” from which some of the properties of classical graph theory can be observed, along with scaling properties. Shirazi et al. (2009) regard particular binned values in a time series as node identifications, and represent the transitions from each time point to the next as an internodal link with probabilities derived from the original series (Shirazi et al., 2009). These methods begin with a time series of a single variable. The target in this paper is distinct in two ways. First, each moment in the fMRI time series is a vector of ∼96,000 continuous variables, while the methods just mentioned begin from time series of one variable. More important, in the application here the goal is not the recovery of a physical network, but rather a compact representation of dynamical (temporal) properties of the image series. We move from one temporal series to another, in effect reducing the dimensionality of time series data. (Accordingly, other methods for dimensionality reduction could also be applied, e.g., Principal component analysis, Independent component analysis, Cluster analysis, among others). The application of graph theory probes the sequence of images as an oscillation among distinct themes (continuing with fully temporal descriptive language).

### Thematic (Temporal) Profiles

Following the application of these techniques, we can consider time series as dynamic temporal profiles, rather than as the expression of fixed spatial networks. Methods going forward, thus, are somewhat novel. This section will introduce them, along with their rationale.

We consider three features of the data sets: repetition, rhythm, and harmony. These are nested: Where repetitions recur with a constant interval between them, there is rhythm. Where there are multiple rhythms and the frequencies of rhythmic repetitions are in integer relations to one another, there is harmony.

#### Repetition

The modular analysis decomposed the time series of global brain activity patterns into a small number of themes, reducing the experiment to a sequence of themes with various durations and alternations. We examined short image sequences (motifs) to see if particular sequences repeat over the image series. We considered sequences of length n, where n ranged from 4 to 11 s. Each sequence (1:n, 2:n + 1, etc.) was compared to all other sequences in each subject’s thematic profile, and exact matches counted.

In the initial analysis, these counts were compared with similar analyses of thematic profiles derived from 100 surrogate association matrices for each subject, as described above. For each sequence length (from 4 to 11 s), repetitions in the data were compared with 100 surrogate data profiles using a one-tailed *t*-test, corrected for multiple comparisons. Then these measures were compared between the two stimulus conditions, rest and movie viewing.

#### Rhythm

Even in domains where rhythm seems apparent, like music, an algorithm for rhythm detection can be elusive – computers can find the beat only very imperfectly (Sethares, 2007). The noisy signals of fMRI are even more difficult, compounded by the absence of intuitive or perceptible rhythms in the data. Here we deploy a continuous measure of “rhythmicity,” understood as the tendency for events that repeat to be separated by a constant interval. It follows from the analysis of repetition, just described. As the repetitions were counted, the intervals between each occurrence of any repeating sequence (motif) as each length (from 4 to 11 images) were also recorded. Where motifs recur more than twice, we compare the intervals between repetitions. If these intervals are equal, then that motif is recurring rhythmically. The presence of these congruent intervals as a proportion of all intervals then serves as one index of rhythmicity. Note that as certain intervals recur more frequently, then other intervals become relatively more rare. This tilt toward rhythmicity is therefore reflected in the standard deviation of the set of numbers of occurrences of each interval. This value can then be compared to standard deviations of random surrogates derived from null network variations generated for each subject. These values then are compared across the two experimental conditions.

#### Harmony

The search for harmony rests on a more tentative approach. A harmonic signal essentially comprises power at a fundamental frequency and/or at integer multiples of that fundamental. Together these higher frequencies form the harmonics of the fundamental. (These are also called overtones or partials). A signal with this spectral structure, then, is harmonic. In principle, harmonicity is easy to detect: the peak amplitude frequencies should be separated in frequency by constant differences. However, with these data neither the fundamental nor the harmonic frequencies are known, and certainly not apparent from the noisy Fourier spectrum. Instead, we exploit the rhythm information just collected: the repeating intervals are easily converted into frequencies, and thus the histogram of intervals transforms into the histogram of frequencies. In effect, this is an alternative form of signal spectrum (not based in Fourier analysis), where numbers of occurrences of each interval converts to amplitude at each frequency.

**FIGURE 2:**
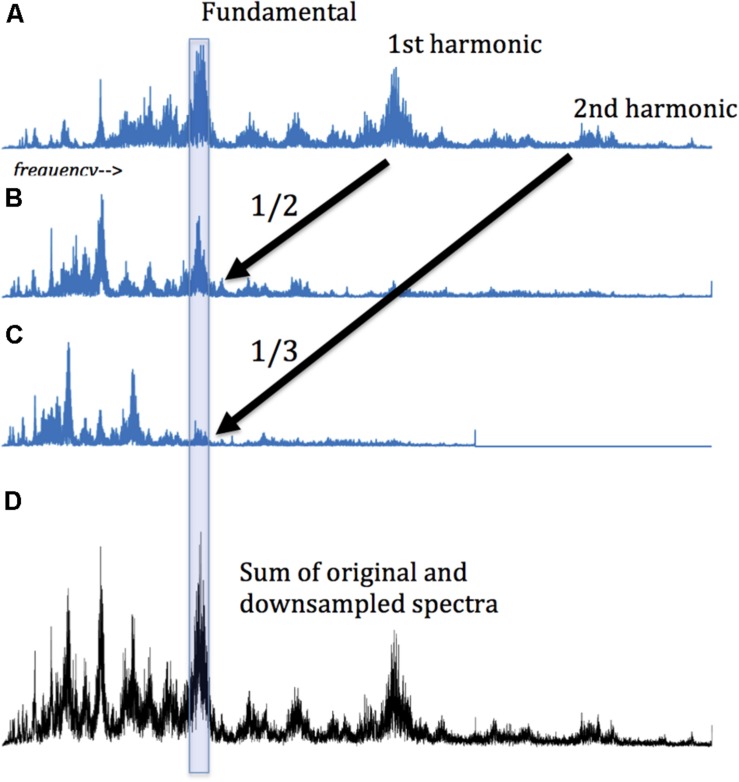
Detecting harmonic signals: A harmonic signal is composed of sinusoidal “partials” whose frequencies are integer multiples of a fundamental frequency. Accordingly, if a harmonic signal spectrum is compressed to one half its original length (i.e., downsampled by 2, taking every other point), then the fundamental and the 1st harmonic will occur at the same point in the original and downsampled spectrum. Their sum thereby amplifies the presence of the harmonic partial. As this process is repeated for successive downsamples, harmonic partials are increasingly amplified. The presence of amplified peaks is thus the marker of harmonic oscillators. **(A)** An original spectrum of frequencies and amplitudes from Fourier analysis of choral singing. **(B)** The same spectrum, downsampled by 2. The 1st harmonic now coincides with the fundamental. **(C)** The orginal spectrum, downsampled by 3. The second harmonic now corresponds with the original fundamental and the 1st harmonic. **(D)** Summing the original and the two downsamplings. Harmonicity is apparent in the sharp peaks in the summation. Note that the summed spectrum no longer represents the original frequency gradient, being is a mix of different frequencies at every point. The original fundamental frequency cannot be recovered from the summation, but we can determine that the fundamental is some integer multiple of the main summed peak. The same conclusion applies to the other peaks in **(D)**, some of which could be fundamental frequencies, while some might be subharmonics of a higher frequency with a greater amplitude, and some might represent higher harmonics of a lower frequency partial.

Then, we adapted the amplitude spectra to amplify the hidden harmonics. Specifically, the spectrum for each subject was downsampled by factors of 2 through 7, and the resultant vectors added to the original. (Downsampling decreases the sampling rate by integer factors. For example, a vector downsampled by a factor of two comprises every second element of the original). The downsampling of a harmonic signal spectrum preserves peaks at the same point in each downsampling. (In effect, each harmonic peak is moved left by an integer factor, so lower and higher harmonics coincide). This is shown schematically in Figure 2. Adding the original and downsampled vectors amplifies the magnitude of the harmonics. This method is similar to “harmonic product spectrum” methods for pitch detection (Cuadra et al., 2001), with the difference that here we sum, rather than multiply, the downsampled vectors. Using this method a maximum value for the summed vectors (original and downsampled transforms) can be calculated. (The position in the vector of this maximum is often interpreted as the fundamental frequency, but this is not necessary for the present analysis). Here we are interested in the maximum magnitude of this compounded amplitude. We identify the presence of harmonics by comparing amplitude at each point in the summed/downsampled vectors to similar points in 100 surrogate datasets, using a one-tailed *t*-test, corrected for multiple comparisons (Bonferroni method). The technique is applied to sequences of each tested length (4 through 11 images). In effect, this analysis considers repeating sequences as oscillators, and groups oscillators by the length of the sequences that repeat. Thus, we cast the net broadly in the hopes that harmonic oscillation can emerge from the background of the inharmonic. These values were compared in the two experimental conditions.

Several standard approaches are the obvious foils: These derive from Fourier analysis and include Wavelet decomposition and measurements of phase synchrony. Fourier analysis construes a signal as a superposition of sinusoids at various frequencies and phases. Or in other words, the basis set for Fourier analysis is a series of sine/cosine functions. Periodic signals are built on that basis, stretched and slipped along the time axis. The second approach takes a brief basis function (the “mother wavelet”), stretches it to various lengths, and matches it to the target signal at every time point. The wavelet decomposition thus construes the signal as a moment by moment superposition of the chosen basis functions, something like a short-segmented or “windowed” Fourier analysis. Phase synchrony between two signals is calculated from instantaneous phase measurements (Lachaux et al., 1999; Varela et al., 2001; Laird et al., 2002; Glerean et al., 2012).

The methods in this study, in contrast to Fourier and Wavelet approaches, make fewer assumptions about the basis function to be tested against the target signal (in this case, a thematic profile). We use segments of the thematic profile itself as basis functions, and analyze the entire signal against each of the thematic profile segments. Thus, every short sequence extracted from the signal is tested along a sliding window as a potential basis function for the whole signal itself. Thus, multiple basis functions are tested, derived from the data itself. For each sequence, we measure its repetition, and from the timing of repetitions we calculate rhythm and harmony. As in the initial parcellation, significance is tested via the permutations derived from the null networks. In this study, the segments tested ranged from four to eleven images (seconds). This window was selected because, in general, the analysis is computationally intensive, requiring some selectivity in what can be feasibly explored. Sequences of shorter than 4 s repeated densely in both the data and the permutations, rendering the comparison moot. At greater than 11 s, repetitions occurred only rarely, attenuating the comparison with the null permutations.

The measurement of rhythm follows a similar strategy, namely, examining the intervals between repetitions of repeating sequences. That periodicity in turn directly determines frequency of the revealed rhythms. These can be tested for harmonic relations, as described above. In general, then, the methods here are both open-ended and data-driven, resting on the sequences that occur in the data, and therefore afford more opportunities for discovering temporal regularities even if transient. In contrast, both Fourier analysis and Wavelet analysis make assumptions about the basis functions. For the FT this is of course the sine function; Wavelets can have many different shapes, but in all cases the analyst specifies the wavelet prior to the analysis. Arguably these assumptions could miss regularities that the methods here might detect. On the other hand both of the standard methods use basis functions stretched to various scales, and so in this sense are more receptive to regularities at multiple scales than the more constrained methods of this study.

Phase synchrony is a powerful marker of functional relatedness (Varela et al., 2001; Laird et al., 2002; Buzsáki and Draguhn, 2004; Glerean et al., 2012; Gupta and Chen, 2016; Gravel et al., 2018). However, it too rests on an *a priori* decision, namely the band-pass filtering of the target signals, necessary for determining instantaneous phase. The methods in the current study identify a range of frequencies involved in multiple rhythms and thus multiple harmonic relationships. Once again, the data is doing the driving.

One final rationale for the methods here is the analogy with music. The concepts of rhythm and harmony employed here are strict analogs of standard usage in musicology. If there is something to be made of the comparison of brain activation and music, these are among the measures we would hope might apply (Lloyd, 2011, 2013).

In summary, the stages of the analysis are these: The starting point is the full pattern of all voxels for each of 900 brain images (for each subject, in two experimental conditions). These are grouped according to a modularity algorithm into themes. The resultant vector, or thematic profile, is the basis for subsequent analysis. To measure repetition, we examine short excerpts or sequences (motifs) drawn from each thematic profile, separately considering all sequences from 4 to 11 s in length, counting the number of repeated occurrences of each sequence. To measure rhythm, we examine the intervals between repetitions of repeating sequences, counting the number of times specific inter-sequence intervals occur. To measure harmonicity, we examine the frequency of rhythmic repetitions, using the downsampling proceedure described above to identify integer ratios between frequencies.

## Results

In this study, we observe the presence of the temporal counterpart to modularity (see Table 1). Within and across subjects, two widely used measures of modularity agreed that the temporal connectome has a modular architecture. Following the Louvain group method, the modularity statistic, summarizing the degree to which the network can be subdivided into groups with high ingroup similarity and low outgroup similarity, averaged 0.1740 (SD 0.05) for the movie-viewing scans and 0.1905 (SD 0.05) for the rest-state scans. Randomized null networks averaged 0.1206 (SD 0.0261). In comparative terms, subject data/surrogate data, the subjects are approximately 1.5 times greater in this statistic than the surrogates. Adjusting for multiple comparisons, 96% of subjects in the movie viewing condition displayed significant modular organization along the time dimension, and 94% in the rest condition. 99% displayed significant modularity in at least one of the two conditions. Pivoted toward time, these module-analogs are called themes. Analysis identified 7.5 and 7.2 themes on average, respectively, in each subject in the two conditions, movie and rest, compared to approximately 12 themes in randomized surrogates. These fundamental observations indicate that the progression of themes is structured, analogous to the modular architectures discovered when graph theory is applied to spatial networks.

**TABLE 1:** Global modularity measures for brain images collected during two experimental conditions.

**Global modularity measures, 180 Ss × 900 images**	**Movie**	**Rest**
*N*	180	180
Mean modularity (*q*) ± SD	0.1740 (0.0546)	0.1905 (0.0542)
(Mean surrogate data)	0.1143 (0.0297)	0.1269 (0.0261)
% significant subjects	96	94
Modal node (theme) count	5	4
Surrogate modal node (theme) count	9	9

*The modularity statistic (*q*) was computed using the Louvain group method (Blondel et al., 2008). Most subjects displayed community-grouping structures over time among recurrent themes, as assessed through contrasts with modularity measures derived from null networks (Rubinov and Sporns, 2010). Actual data tended toward network reconstructions with around five distinct themes. The surrogate models oscillated among more distinct themes.*

Subsequent questions, then, probe the origin and structure of the apparent temporal dynamics, collected in Table 2. Repetition was measured by simply comparing all the subsequences of 4–11 images in each thematic profile, and counting exact matches throughout the profile. This analysis found highly significant repetition for at least some subjects at all of these durations. Overall, during the rest condition 81% of subjects displayed at least one sequence with repetitions greater than the surrogates. On average, each subject displayed significant repetition for 6 sequence lengths. The sequence length exhibiting repetition in the largest subset of subjects was 6 s. During the movie viewing condition, 87% of subjects showed repetition for at least one sequence length; on average, each manifested repetition for 6 different sequence lengths. Sequence lengths of 6 or 7 s were the most frequent repeaters.

**TABLE 2:** Repetition, rhythm, and harmony.

**Repetition, Rhythm, and Harmony**								

**Passive movie viewing**								

	**Sequence length (s)**							
	**4**	**5**	**6**	**7**	**8**	**9**	**10**	**11**
**Repetition**								
% of Ss with significant values	67	75	78	81	79	82	78	77
Repeats of themes/length of image series	1.70	1.09	0.73	0.48	0.33	0.23	0.17	0.12
Std	0.78	0.54	0.41	0.31	0.24	0.19	0.15	0.12
**Rhythm**								
% of Ss with significant values	76	85	85	86	82	72	63	59
Rhythmicity: std of significant interval counts	1.60	1.48	1.26	1.06	0.86	0.68	0.55	0.47
Mean difference from random surrogates	0.78	0.80	0.71	0.63	0.57	0.47	0.39	0.36
**Harmony**								
% of Ss with significant values	99	100	99	98	97	90	87	84
Median # of significant freqs per S	48.0	65.0	62.5	57.5	50.0	44.0	37.5	34.5
Harmonicity: mean ratio data vs. surrogates, power at downsampled frequencies	3.40	12.95	15.34	8.21	6.60	7.17	8.35	8.48
± SEM	0.08	0.88	0.90	0.30	0.20	0.21	0.28	0.33
**Rest**								
**Repetition**								
% of Ss with significant values	61	68	72	73	69	67	63	61
Repeats of themes/length of image series	1.82	1.18	0.78	0.50	0.34	0.23	0.16	0.11
Std	0.84	0.70	0.49	0.35	0.29	0.23	0.18	0.14
**Rhythm**								
% of Ss with significant values	78	84	87	83	72	67	56	53
Rhythmicity: std of significant interval counts	1.71	1.55	1.34	1.05	0.80	0.64	0.48	0.39
Mean difference from random surrogates	0.71	0.75	0.67	0.56	0.49	0.43	0.32	0.28
**Harmony**								
% of Ss with significant values	99	100	100	100	97	96	93	92
Median # of significant freqs per S	42.00	53.00	56.00	54.00	47.00	43.00	35.00	30.00
Harmonicity: mean ratio data vs. surrogates, power at downsampled frequencies	2.09	10.84	10.91	12.90	13.54	8.60	6.42	6.71
± SEM	0.05	1.23	0.59	0.57	0.79	0.41	0.22	0.26

*The temporal connectome as it arises in the brain is characterized by high degrees of repetition, the presence of regular intervals between repetitions (i.e., rhythm), and the integer multiple relations among the frequencies of rhythms, as determining by the downsampled sum of spectra described in the text and Figure 2.*

Rhythmicity was measured by examining the intervals between repetitions and counting the number of recurrences of each interval. As with the repetitions, this was separately examined for sequences of lengths 4 through 11 s. These tabulations were compared with similar tabulations for 100 surrogate data sets, and significant deviations recorded (as always, correcting for multiple comparisons). Overall 88 and 91%, respectively, of movie and rest condition subjects showed significant rhythmicity at least one sequence length. Sequences of 6 or 7 s were most often rhythmic, displayed by around 86% of subjects in both conditions.

Harmonicity was measured by summing the original and six downsampled spectra for each subject. By shrinking the spectra by an integer factor, the downsampling preserves peaks in relationships of integer multiples – i.e., harmonics. (Due to the downsampling, the peaks of the summed downsampled spectra cannot be assigned to specific frequencies). This test yielded highly significant harmonics for all subjects in the two conditions. Sequence lengths of 4–7 s were harmonically organized in nearly all subjects, with all subjects viewing movies displaying harmony for 5 s sequences, and all subjects in the rest condition displaying harmonics for sequences of 5, 6, and 7 s. In both conditions over 40 specific peaks exceeded baseline surrogate measures in both conditions.

## Discussion

### What Now? What’s Next?

Very generally, animal brains face a dual computational demand: they must sense (and interpret) what is immediately present; and they must predict what will happen next (over a future from milliseconds to hours to years) (Friston and Stephan, 2007; Clark, 2013, 2016; Hohwy, 2013). In computational terms, all animals need a capacity to continuously maintain representations of past and future environmental (and bodily) conditions, while at the same time continuously refreshing, updating, and modifying these representations as new information arrives.

This sketch of cognition is subject to three general constraints: First, to be useful, information that is generated at one source must be available to modify information elsewhere. Global availability leads to the second constraint: For information to be effectively integrated across the brain, it must be transmitted with minimum confusion. In effect, channels must converge and diverge without crosstalk. Third, all this temporal mixing and matching must work quickly, to keep up with a dynamic world.

### Oscillation, Information Broadcasting, and Maintenance

How might repetition, rhythm, or harmony enable these computational ends? Oscillations are everywhere at frequencies from less than 1 to 150 Hz (Biswal et al., 1995; Linkenkaer-Hansen et al., 2001; Buzsáki and Draguhn, 2004; Buzsáki, 2006; Zuo et al., 2010; Kopell et al., 2014), and most likely originate in neural activity (Zuo et al., 2010). Along with their observation we find a cornucopia of proposals for their function. Many of these posit interactions among oscillations at different frequencies, where frequency bands have distinct functions (Glassman, 1999; Onslow et al., 2011; Aru et al., 2015; Wiener and Kanai, 2016; Lundqvist et al., 2018; Zhang et al., 2018). For example, Friston et al. (2015) have proposed that theta and gamma oscillations signal from the periphery up while beta oscillations provide feedback from the top down in the visual system. Other researchers propose that oscillations perform a gating function, for synchronizing signals (Buzsáki and Draguhn, 2004; Fries, 2005, 2015; Maris et al., 2016). Canolty et al. (2010) propose that oscillation is the electrophysiological signature of Hebbian cell assemblies at work (Canolty et al., 2010). In general, however, oscillation implies a capacity for information maintenance (Buzsáki and Draguhn, 2004). This may be important for maintaining attention and working memory, among other functions (Buzsáki and Draguhn, 2004; Hipp et al., 2011; Aru et al., 2015; Fries, 2015; Gregoriou et al., 2015; Gupta and Chen, 2016). Instantaneous coupling of signals (“microstates”) are another variation on the communicative role of oscillations (Schack, 2004; Dimitriadis et al., 2013). These are generally derived from EEG data, with the exception of Ville et al. (2010) and Hipp et al. (2011), who found scale-free dynamics including the frequencies observed through fMRI, and Zuo et al. (2010) who explored frequency bands below 0.1 Hz, to identify regions of the brain where oscillations within particular frequency bands exhibited greater amplitude.

### Rhythm

The rhythms described in this study are particularly apt candidates for temporal holding patterns, in that the methods here define the elements in rhythmic repetition as sequences of thematic moments. What is repeating is itself a temporally extended pattern (4–11 s), drawn from an alphabet of around seven themes. The many rhythms found in both the rest and movie conditions invite a more detailed study of these patterns, in addition to research into their frequency of oscillation (Zuo et al., 2010).

### Harmony

Among the many discussions of oscillations in the brain, discussions of harmonic relationships among frequencies are rare. [Atasoy et al. (2016) probes spatial frequencies in harmonic relations, but not temporal harmonics]. Yet harmonic partials are rampant in the data in the present study – at least 30 partials were discovered in the frequencies of sequence occurrence at all sequence lengths. What could be the functional significance of this widespread observation?

**FIGURE 3:**
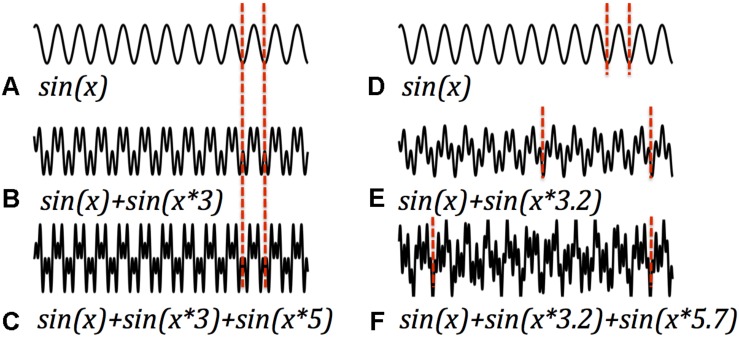
Harmonic and inharmonic periodic signals. Left panels display a basic sinusoidal signal **(A)** and two signals with the fundamental frequency of **(A)** plus one harmonic **(B)** and two harmonics **(C)**. The period of each cycle is demarcated with vertical dashed lines. In the harmonic signals the period remains the same as packages of different harmonics are added. Right panels display two inharmonic signals **(E,F)** which result when the added signals are not integer multiples of the fundamental (The basic sinusoid signal **(A)** is recapitulated in panel **(D)**, for ease of comparison.). One effect of inharmicity is the stretching of periods. Multiple frequencies when added are most efficiently encoded in harmonic signals.

A speculative argument could begin with functional distinctions between the stages of signal processing in any system, the brain included. A full mechanistic account of such a system must explain how signals are generated, how they are transmitted, and how they are received/interpreted. Abundant research explores how neuronal oscillations are generated; a fairly large literature considers how signals propagate over space and time; but there is little consideration of how a received signal is processed. Fourier analyses are computationally intensive, and require many signal samples to be precise – it seems unlikely that the brain computes in this way. In a periodic signal, however, the minimum interpretable packet is one cycle. Accordingly, in principle the fastest processing is most feasible when cycle time, the interval between repetitions of the periodic signal, is shortest. In general, harmonic signals offer a useful combination of multiple superimposed frequencies and short cycle times. This follows from the definition of harmonics, which are signals whose frequencies are in integer ratios, but might also be illustrated with an example. The left panel of Figure 3 illustrates different harmonic signals, the pure signal at one frequency, sin(*x*), and several composite signals, sin(*x*) + sin(*x* × C) where *x* is a monotonic vector (of time points) and C is an integer. Accordingly, the composite signals on the left side of the figure are harmonic signals of various lengths, with the same fundamental frequency. For each, one cycle is bracketed. The right panel presents examples of inharmonic signals and their cycle times. It is apparent that the harmonic signals have the shortest cycles, as is indeed implied by the integer relationships of their frequencies. Thus, if a signal is composed of multiple frequencies, the smallest package (quickest, easiest, most efficient) that delivers all the frequency information employs frequencies in harmonic relationships. The example introduces just two harmonic partials, but this hypothetical computational process can accommodate more complex harmonic signals as well. The presence and absence of harmonics afford the system a binary code, albeit one of modest capacity. Such a system gets off the ground without Fourier analysis, and packages its message in a minimum interval (Glassman, 2000).

Harmonic signals, in short, afford rapid “unpacking” by their receivers, a property that might make them adaptive for natural selection.

Could the brain implement a computational process that can extract harmonics from a mixed signal? Encouragement comes from the real biological analogy of hearing, which sorts fundamentals and overtones through a combination of cochlear shape and specialized sensory neurons. It does so quickly, but not in a single cycle. Glassman considered harmonic information at the timescale captured by EEG, proposing that short term memory with its famous capacity limitation might be embodied in a harmonic resonance of a complex marker or cue for each memorized item (Glassman, 1999). In each octave of any resonator the number of subharmonics is limited [subharmonics are frequencies of higher harmonics dropped from their octaves (halved repeatedly) into a single octave]. He hypothesized that the harmonics could keep separate the items while binding them in a stable system of resonances, amenable to extraction at the time of recall. In the current paper the time resolution of fMRI limits the analysis to very low frequencies. A single cycle affords ample neural computation time. Indeed, it’s most likely that any real neural process that exploits harmonics is operating at higher frequencies, leaving only subharmonics in the cycles discernible to fMRI (Buzsáki, 2006).

How might signal analysis work at the neural level? Timing in many animals may be supported by harmonic properties of the interaction of oscillations from particular brain regions (Gupta and Chen, 2016). The “striatal beat frequency” model of timing posits multiple oscillators at frequencies in harmonic relationships (Matell and Meck, 2004; Buhusi and Meck, 2005; Merchant et al., 2011; Kononowicz and van Wassenhove, 2016). For example, suppose there are three distinct oscillators with periods of 2, 3, and 5 s. At various moments their oscillations will coincide. The 2 s and 3 s oscillations reinforce at every 6 s, while 2 and 5 converge every 10 s. All three reinforce every 30 s. To time intervals of 6, 10, or 30 s a system needs simply to detect these convergences. Timing in this example is a punctate response. Temporal perception emerges when we imagine continuous relationships among harmonics. If the “beats” are rising and falling gradients, as might emerge from a time-varying harmonic signal, their mix could provide continuous temporal information.

**FIGURE 4:**
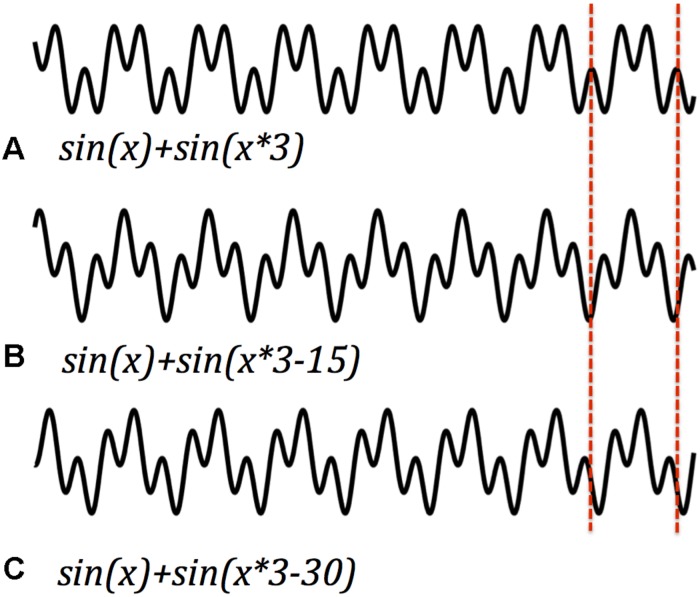
Harmonic signals with the same frequency but different phase offset differ in their wave form for each cycle (e.g., **A–C**). Phase offsets and wave forms. Harmonic signals with the same frequency but different phase offset differ in their wave form for each cycle. Dashed lines demarcate one cycle. Phasic information could be recovered from relative amplitude peaks within a single cycle.

Frequency is only part of the information that could be encoded by harmonics. Harmonic signals also differentiate by the relative phase of their component frequencies, which is the offset of the zero-crossings of their cycles. Since amplitude is additive from moment to moment in any signal, in-phase and out-of-phase signals have very different overall shapes (despite similar spectra). This offers another feature with a capacity to carry temporal information (Gupta and Chen, 2016; Hakim and Vogel, 2018). The phase of a periodic signal is set at the origin of the signal, or (more likely) reset by an event that interrupts continuing oscillation. If different events reset different signals at different frequencies, and if those frequencies are harmonically related, then the ongoing signal encodes the interval between the initiating events. Figure 4 illustrates an example. Each is the sum of the same fundamental and first harmonic. However, in each panel the origin of the harmonic sinusoid has been time-shifted by a different interval. As in the earlier examples, the cycle time remains the same, and the overall structure of peaks within each period. But the relative magnitude of the peaks shifts with the mismatched phases. One cycle of a harmonic signal, it seems, can signal phasic differences, another available and quick vehicle for usable information. I’ve suggested that the event of initiation of a resonant wave could be what determines its phase. If that’s so, then the package carries a rough representation of sequence. Once again, harmonics can be added, each with a different phase (see also Onslow et al., 2011; Maris et al., 2016; Zhang et al., 2018).

To summarize this discussion, the computational constraints of temporal context, separability of signals, and speed might be efficiently met in a resonating system that is wired for rhythm and harmony.

### Limitations and Future Directions

Recent literature has emphasized the importance of confirming the reliability of the many measures typical of FMRI research (Zuo and Xing, 2014; Poldrack et al., 2017; Zuo et al., 2019a, b). This has not been undertaken in this paper. Therefore, the actual statistical power of the analysis awaits further study. Meanwhile, however, several features of the data and its analysis indicate that the main results of this study do rest on reliable observations. First, the effects described are large, as is the size of the data set, with 180 subjects examined. For example, the modularity measure used here, when compared to the 100 null networks for each subject and task, has an effect size greater than 1 by Hedges’ g (Hedges, 1981). These observations are consistent across the two tasks examined, similar to the report of O’Connor et al. (2017) comparing four experimental conditions. More important, the Human Connectome Project has had replicability as a major goal (Marcus et al., 2013), and its reliability is supported in Termenon et al. (2016). Scanning at 7 Tesla, using standardized preprocessing pipelines, and the application of cortical surface coordinates to localize brain activity all increase confidence in the consistency of the scans (but see Cremers et al., 2017; Poldrack et al., 2017; Smith and Nichols, 2018). Studies of the reliability of HCP subjects have been less frequent, although the HCP records more than 500 phenotypic features for each subject, again indicating the care the researchers are bringing to their task. Nonetheless the analysis here should be regarded as provisional, pending a future examination of this issue.

## Conclusion

### Temporal Processing in the Brain

This paper offers an initial attempt to outline novel aspects of temporality in the human brain, at the low temporal resolution afforded by fMRI. The first step was to create a matrix of relationships among moments, an association matrix determined by similarity of patterns. This immediately revealed that the brain patterns are not simply counting off the seconds, where each image is most similar to its nearest temporal neighbor. Instead, the brains of our subjects moved among a small number of states. These clusters can be established by many different methods (Lloyd, 2002, 2004, 2012). Here we employed graph theoretic measures of modularity to outline the stable clusters we’ve called themes. The “network” discovered in this way is a sequential alternation of themes, a thematic profile. This served as a first approximation of temporal processing analyzed independently from the general framework of Fourier and wavelet analysis.

The observation of modular temporal structure motivated a search for intrinsic temporal anatomy, regularities within the thematic structures projected by graph theory. There are many avenues of exploration possible. Here, we looked at the regular manifestations of repetition, rhythm, and especially harmony, a distinctively useful configuration of oscillations that seems to exploit discernible features that only harmonic signals possess. The analysis here suggested that there are harmonic relationships underlying the oscillations of the thematic profiles; their computational uses suggest that harmonic signaling might be a useful adaptation. Harmonic signals provide short repeating temporal motifs from which separate frequencies can be extracted. The motifs resonate at their fundamental frequency and within each period harmonics oscillate in regular patterns. These patterns are further modified by the relative phases of the component frequencies.

In this package of harmonics we may discern a basic structure of temporality. It is a resonating holding pattern which carries information originating before the interval of each repetition. The broad similarities in the temporal properties in two very different experimental conditions, movie viewing and rest, imply that the observed rhythms and harmonies are fundamental to informational processes in the brain. From this vantage point we can’t determine if the global brain patterns we observe originate from separate sources with distinct frequency profiles, or from a single harmonic resonator, but we can observe the relationships among the frequencies that can be extracted. Moreover, in this analysis we haven’t examined relative phase for the extracted harmonics, but here too this information could come from a single global source or multiple sources. The answers to these questions would pivot back toward the spatial, for example, measuring the amplitudes of oscillations in various brain regions (Zuo et al., 2010). But strictly within the temporal realm, brain dynamics are orderly and perseverating. The observations here suggest a human capacity to spread out from the immediate present tense of sensation, toward an overall temporal landscape. The brains examined here show signs of a present inflected by a past that resonates and possibly holds information about sequence and interval, and thus can also encode expectations of the immediate future. There is, of course, much that is speculative here; these proposals are offered as a starting point for further study.

### The Music of Thought

Certain concepts seem apt for redeployment in the study of temporality: theme and modularity, repetition, rhythm, and especially harmony. These of course are familiar through music. In other works, I’ve suggested that the analogies between brain dynamics and musical form should be taken literally, or at any rate as literally as language in the hypothetical “language of thought” (Lloyd, 2011). Musical concepts have one common feature that makes them especially useful here: time is essential to all of them, and so they are properties appropriate to the observations following the pivot from space to time (Lloyd, 2013). Such concepts *could* apply, but it is an empirical question whether they do apply. Are there in fact rhythm, theme and refrain, and harmony in fMRI signals? In the experiment reviewed here, the answer is a (tentative) yes. Indeed, the fMRI analyses here point to the pervasive presence of repetition, rhythm, and especially harmony. Among human artifacts, only music approaches this density and structure of repetition (Huron, 2014). In sharp contrast, these properties are at best weakly present in language, which has often been proposed as the model for cognition and ultimately brain function (Fodor, 1979; Lloyd, 2011).

One attractive topic for further exploration is the scaling behavior of the rhythms observed here (He et al., 2010; Kello et al., 2010; Ville et al., 2010; Hardstone et al., 2012). Since frequency here has a novel definition, scaling behaviors would require a distinct test, a topic for future study [especially since music exhibits well-known scaling laws (Manaris et al., 2005; Lloyd, 2011; González-Espinoza et al., 2017)].

The literal connection to music may seem implausible. After all, music is a cultural artifact and art form seemingly incidental to the serious business of survival and reproduction – “auditory cheesecake,” in Pinker’s (1997) memorable phrase. But several considerations suggest that sidelining music is a mistake. In many ways music is fully parallel to language in its intimacy with the human condition: Music is universal to human cultures (Patel, 2010); World musical systems almost universally share certain features, including the use of scales and limited rhythmic patterns (Huron, 2014); Music is old. [The oldest instrument, a carefully crafted flute, was made more than 40,000 years ago (Higham et al., 2012)]. Music is potentially advantageous for social cohesion or sexual selection (Levitin, 2007; Patel, 2010). Music, unlike language, lacks the power to denote specific objects and scenes (Hanslick, 1891; Kivy, 1990; Lloyd, 2011). But what it might represent, by analogy, is the dynamic operation of the brain itself. Musicians may be improvising a model of mind in sound. In that case, musical concepts are not externals brought to bear on brain dynamics, but rather the natural, intuitive expression of that dynamic, a “music of thought.”

## Author Contributions

The author confirms being the sole contributor of this work and has approved it for publication.

## Conflict of Interest

The author declares that the research was conducted in the absence of any commercial or financial relationships that could be construed as a potential conflict of interest.

## Footnotes

1 https://www.humanconnectome.org/

2 https://db.humanconnectome.org

3 https://www.humanconnectome.org/storage/app/media/documentation/s1200/HCP_S1200_Release_Reference_Manual.pdf

4 https://sites.google.com/site/bctnet/

## References

[B1] Allen E. A. Damaraju E. Plis S. M. Erhardt E. B. Eichele T. Calhoun V. D. (2014). Tracking whole-brain connectivity dynamics in the resting state. *Cereb. Cortex N. Y.* 24 663–676. 10.1093/cercor/bhs352 23146964 PMC3920766

[B2] Aru J. Aru J. Priesemann V. Wibral M. Lana L. Pipa G. (2015). Untangling cross-frequency coupling in neuroscience. *Curr. Opin. Neurobiol.* 31 51–61. 10.1016/j.conb.2014.08.002 25212583

[B3] Atasoy S. Donnelly I. Pearson J. (2016). Human brain networks function in connectome-specific harmonic waves. *Nat. Commun.* 7:10340. 10.1038/ncomms10340 26792267 PMC4735826

[B4] Biswal B. Yetkin F. Z. Haughton V. M. Hyde J. S. (1995). Functional connectivity in the motor cortex of resting human brain using echo-planar MRI. *Magn. Reson. Med.* 34 537–541. 10.1002/mrm.1910340409 8524021

[B5] Blondel V. D. Guillaume J.-L. Lambiotte R. Lefebvre E. (2008). Fast unfolding of communities in large networks. *J. Stat. Mech. Theory Exp.* 2008:10008. 21517554

[B6] Buhusi C. V. Meck W. H. (2005). What makes us tick? Functional and neural mechanisms of interval timing. *Nat. Rev. Neurosci.* 6 755–765. 10.1038/nrn1764 16163383

[B7] Bullmore E. Sporns O. (2009). Complex brain networks: graph theoretical analysis of structural and functional systems. *Nat. Rev. Neurosci.* 10 186–198. 10.1038/nrn2575 19190637

[B8] Buzsáki G. (2006). *Rhythms of the Brain.* Oxford: Oxford University Press.

[B9] Buzsáki G. Draguhn A. (2004). Neuronal oscillations in cortical networks. *Science* 304 1926–1929. 10.1126/science.1099745 15218136

[B10] Calhoun V. D. Miller R. Pearlson G. Adali T. (2014). The chronnectome: time-varying connectivity networks as the next frontier in fMRI data discovery. *Neuron* 84 262–274. 10.1016/j.neuron.2014.10.015 25374354 PMC4372723

[B11] Canolty R. T. Ganguly K. Kennerley S. W. Cadieu C. F. Koepsell K. Wallis J. D. (2010). Oscillatory phase coupling coordinates anatomically dispersed functional cell assemblies. *Proc. Natl. Acad. Sci. U.S.A.* 107 17356–17361. 10.1073/pnas.1008306107 20855620 PMC2951408

[B12] Chakravartty A. (2017). *Scientific Ontology: Integrating Naturalized Metaphysics and Voluntarist Epistemology.* Oxford: Oxford University Press.

[B13] Clark A. (2013). Whatever next? Predictive brains, situated agents, and the future of cognitive science. *Behav. Brain Sci.* 36 181–204. 10.1017/S0140525X12000477 23663408

[B14] Clark A. (2016). *Surfing Uncertainty: Prediction, Action, and the Embodied Mind.* Oxford: Oxford University Press.

[B15] Cremers H. R. Wager T. D. Yarkoni T. (2017). The relation between statistical power and inference in fMRI. *PLoS One* 12:e0184923. 10.1371/journal.pone.0184923 29155843 PMC5695788

[B16] Cuadra P. D. Master A. S. Sapp C. S. (2001). “Efficient pitch detection techniques for interactive music,” in *Proceedings of the International Computer Music Conference*, (San Francisco, CA).

[B17] Dimitriadis S. I. Laskaris N. A. Tzelepi A. (2013). On the quantization of time-varying phase synchrony patterns into distinct functional connectivity microstates (FCμstates) in a multi-trial visual ERP paradigm. *Brain Topogr.* 26 397–409. 10.1007/s10548-013-0276-z 23443252

[B18] Epskamp S. Fried E. I. (2018). A tutorial on regularized partial correlation networks. *Psychol. Methods* 23 617–634. 10.1037/met0000167 29595293

[B19] Fallani F. D. V. Richiardi J. Chavez M. Achard S. (2014). Graph analysis of functional brain networks: practical issues in translational neuroscience. *Philos. Trans. R. Soc. B Biol. Sci.* 369:20130521. 10.1098/rstb.2013.0521 25180301 PMC4150298

[B20] Fodor J. A. (1979). *The Language of Thought.* Cambridge, MA: Harvard University Press.

[B21] Fries P. (2005). A mechanism for cognitive dynamics: neuronal communication through neuronal coherence. *Trends Cogn. Sci.* 9 474–480. 10.1016/j.tics.2005.08.011 16150631

[B22] Fries P. (2015). Rhythms for cognition: communication through coherence. *Neuron* 88 220–235. 10.1016/j.neuron.2015.09.034 26447583 PMC4605134

[B23] Friston K. J. Bastos A. M. Pinotsis D. Litvak V. (2015). LFP and oscillations—what do they tell us? *Curr. Opin. Neurobiol.* 31 1–6. 10.1016/j.conb.2014.05.004 25079053 PMC4376394

[B24] Friston K. J. Stephan K. E. (2007). Free-energy and the brain. *Synthese* 159 417–458. 19325932 10.1007/s11229-007-9237-y PMC2660582

[B25] Girvan M. Newman M. E. J. (2002). Community structure in social and biological networks. *Proc. Natl. Acad. Sci. U.S.A.* 99 7821–7826. 12060727 10.1073/pnas.122653799 PMC122977

[B26] Glasser M. F. Coalson T. S. Robinson E. C. Hacker C. D. Harwell J. Yacoub E. (2016). A multi-modal parcellation of human cerebral cortex. *Nature* 536 171–178. 10.1038/nature18933 27437579 PMC4990127

[B27] Glasser M. F. Sotiropoulos S. N. Wilson J. A. Coalson T. S. Fischl B. Andersson J. L. (2013). The minimal preprocessing pipelines for the human connectome project. *NeuroImage* 80 105–124. 10.1016/j.neuroimage.2013.04.127 23668970 PMC3720813

[B28] Glassman R. B. (1999). Hypothesized neural dynamics of working memory: several chunks might be marked simultaneously by harmonic frequencies within an octave band of brain waves. *Brain Res. Bull.* 50 77–93. 10.1016/s0361-9230(99)00090-8 10535328

[B29] Glassman R. B. (2000). A “Theory of relativity” for cognitive elasticity of time and modality dimensions supporting constant working memory capacity: involvement of harmonics among ultradian clocks? *Prog. Neuropsychopharmacol. Biol. Psychiatry* 24 163–182. 10.1016/s0278-5846(99)00096-2 10800741

[B30] Glerean E. Salmi J. Lahnakoski J. M. Jääskeläinen I. P. Sams M. (2012). Functional magnetic resonance imaging phase synchronization as a measure of dynamic functional connectivity. *Brain Connect.* 2 91–101. 10.1089/brain.2011.0068 22559794 PMC3624768

[B31] González-Espinoza A. Larralde H. Martínez-Mekler G. Müller M. (2017). Multiple scaling behaviour and nonlinear traits in music scores. *R. Soc. Open Sci.* 4:171282. 10.1098/rsos.171282 29308256 PMC5750023

[B32] Gravel N. Harvey B. M. Renken R. J. Dumoulin S. O. Cornelissen F. W. (2018). Phase-synchronization-based parcellation of resting state fMRI signals reveals topographically organized clusters in early visual cortex. *Neuroimage* 170 424–433. 10.1016/j.neuroimage.2017.08.063 28867341

[B33] Gregoriou G. G. Paneri S. Sapountzis P. (2015). Oscillatory synchrony as a mechanism of attentional processing. *Brain Res.* 1626 165–182. 10.1016/j.brainres.2015.02.004 25712615

[B34] Gupta D. S. Chen L. (2016). Brain oscillations in perception, timing and action. *Curr. Opin. Behav. Sci.* 8 161–166. 10.1016/j.cobeha.2016.02.021

[B35] Hagmann P. Kurant M. Gigandet X. Thiran P. Wedeen V. J. Meuli R. (2007). Mapping human whole-brain structural networks with diffusion MRI. *PLoS One* 2:e597. 10.1371/journal.pone.0000597 17611629 PMC1895920

[B36] Hakim N. Vogel E. K. (2018). Phase-coding memories in mind. *PLoS Biol.* 16:e3000012. 10.1371/journal.pbio.3000012 30157170 PMC6133377

[B37] Hanslick E. (1891). *The Beautiful in Music; a Contribution to the Revisal of Musical Æsthetics.* London: Novello and Co.

[B38] Hardstone R. Poil S.-S. Schiavone G. Jansen R. Nikulin V. V. Mansvelder H. D. (2012). Detrended fluctuation analysis: a scale-free view on neuronal oscillations. *Front. Physiol.* 3:450. 10.3389/fphys.2012.00450 23226132 PMC3510427

[B39] He B. J. Zempel J. M. Snyder A. Z. Raichle M. E. (2010). The temporal structures and functional significance of scale-free brain activity. *Neuron* 66 353–369. 10.1016/j.neuron.2010.04.020 20471349 PMC2878725

[B40] Hedges L. V. (1981). Distribution theory for Glass’s Estimator of effect size and related estimators. *J. Educ. Stat.* 6 107–128. 10.3102/10769986006002107

[B41] Higham T. Basell L. Jacobi R. Wood R. Ramsey C. B. Conard N. J. (2012). T esting models for the beginnings of the Aurignacian and the advent of figurative art and music: the radiocarbon chronology of Geißenklösterle. *J. Hum. Evol.* 62 664–676. 10.1016/j.jhevol.2012.03.003 22575323

[B42] Hipp J. F. Engel A. K. Siegel M. (2011). Oscillatory synchronization in large-scale cortical networks predicts perception. *Neuron* 69 387–396. 10.1016/j.neuron.2010.12.027 21262474

[B43] Hodge M. R. Horton W. Brown T. Herrick R. Olsen T. Hileman M. E. (2016). ConnectomeDB—Sharing human brain connectivity data. *NeuroImage* 124 (Pt B), 1102–1107. 10.1016/j.neuroimage.2015.04.046 25934470 PMC4626437

[B44] Hohwy J. (2013). *The Predictive Mind.* Oxford: Oxford University Press.

[B45] Huron D. (2014). *Sweet Anticipation: Music and the Psychology of Expectation.* Cambridge: MIT Press.

[B46] Hutchison R. M. Womelsdorf T. Allen E. A. Bandettini P. A. Calhoun V. D. Corbetta M. (2013). Dynamic functional connectivity: promise, issues, and interpretations. *NeuroImage* 80 360–378. 10.1016/j.neuroimage.2013.05.079 23707587 PMC3807588

[B47] Kaiser M. (2011). A tutorial in connectome analysis: topological and spatial features of brain networks. *NeuroImage* 57 892–907. 10.1016/j.neuroimage.2011.05.025 21605688

[B48] Kello C. T. Brown G. D. A. Ferrer-i-Cancho R. Holden J. G. Linkenkaer-Hansen K. Rhodes T. (2010). Scaling laws in cognitive sciences. *Trends Cogn. Sci.* 14 223–232. 10.1016/j.tics.2010.02.005 20363176

[B49] Kivy P. (1990). *Music Alone: Philosophical Reflections on the Purely Musical Experience.* Ithaca: Cornell University Press.

[B50] Kononowicz T. W. van Wassenhove V. (2016). In search of oscillatory traces of the internal clock. *Front. Psychol.* 7:224. 10.3389/fpsyg.2016.00224 26941683 PMC4763057

[B51] Kopell N. Gritton H. J. Whittington M. A. Kramer M. A. (2014). Beyond the connectome: the dynome. *Neuron* 83 1319–1328. 10.1016/j.neuron.2014.08.016 25233314 PMC4169213

[B52] Lacasa L. Luque B. Ballesteros F. Luque J. Nuño J. C. (2008). From time series to complex networks: the visibility graph. *Proc. Natl. Acad. Sci. U.S.A.* 105 4972–4975. 10.1073/pnas.0709247105 18362361 PMC2278201

[B53] Lachaux J.-P. Rodriguez E. Martinerie J. Varela F. J. (1999). Measuring phase synchrony in brain signals. *Hum. Brain Mapp.* 8 194–208. 10.1002/(sici)1097-0193(1999)8:4<194::aid-hbm4>3.0.co;2-c 10619414 PMC6873296

[B54] Laird A. R. Rogers B. P. Carew J. D. Arfanakis K. Moritz C. H. Meyerand M. E. (2002). Characterizing instantaneous phase relationships in whole-brain fMRI activation data. *Hum. Brain Mapp.* 16 71–80. 10.1002/hbm.10027 11954057 PMC6872093

[B55] Levitin D. J. (2007). *This is Your Brain on Music: the Science of a Human Obsession.* New York: Plume.

[B56] Linkenkaer-Hansen K. Nikouline V. V. Palva J. M. Ilmoniemi R. J. (2001). Long-range temporal correlations and scaling behavior in human brain oscillations. *J. Neurosci.* 21 1370–1377. 10.1523/jneurosci.21-04-01370.2001 11160408 PMC6762238

[B57] Lloyd D. (2002). Functional MRI and the study of human consciousness. *J. Cogn. Neurosci.* 14 818–831. 10.1162/089892902760191027 12191448

[B58] Lloyd D. (2004). *Radiant Cool: a Novel Theory of Consciousness.* Cambridge, MA: MIT Press.

[B59] Lloyd D. (2011). Mind as music. *Front. Psychol.* 2:63. 10.3389/fpsyg.2011.00063 21687438 PMC3110500

[B60] Lloyd D. (2012). “Time after time,” in *Being in Time: Dynamical Models of Phenomenal Experience*, eds Edelman S. Fekete T. Zach N. (Amsterdam: John Benjamins), 88–81.

[B61] Lloyd D. (2013). The music of consciousness: can musical form harmonize phenomenology and the brain? *Constr. Found.* 8 324–331.

[B62] Lundqvist M. Herman P. Warden M. R. Brincat S. L. Miller E. K. (2018). Gamma and beta bursts during working memory readout suggest roles in its volitional control. *Nat. Commun.* 9 1–12. 10.1038/s41467-017-02791-8 29374153 PMC5785952

[B63] Manaris B. Romero J. Machado P. Krehbiel D. Hirzel T. Pharr W. (2005). Zipf’s law, music classification, and aesthetics. *Comput. Music J.* 29 55–69. 10.1162/comj.2005.29.1.55

[B64] Marcus D. Harwell J. Olsen T. Hodge M. Glasser M. Prior F. (2011). Informatics and data mining tools and strategies for the human connectome project. *Front. Neuroinformatics* 5:4. 10.3389/fninf.2011.00004 21743807 PMC3127103

[B65] Marcus D. S. Harms M. P. Snyder A. Z. Jenkinson M. Wilson J. A. Glasser M. F. (2013). Human connectome project informatics: quality control, database services, and data visualization. *Neuroimage* 80 202–219. 10.1016/j.neuroimage.2013.05.077 23707591 PMC3845379

[B66] Maris E. Fries P. van Ede F. (2016). Diverse phase relations among neuronal rhythms and their potential function. *Trends Neurosci.* 39 86–99. 10.1016/j.tins.2015.12.004 26778721

[B67] Marrelec G. Horwitz B. Kim J. Pélégrini-Issac M. Benali H. Doyon J. (2007). Using partial correlation to enhance structural equation modeling of functional MRI data. *Magn. Reson. Imaging* 25 1181–1189. 10.1016/j.mri.2007.02.012 17475433

[B68] Maslov S. Sneppen K. (2002). Specificity and stability in topology of protein networks. *Science* 296 910–913. 10.1126/science.1065103 11988575

[B69] Matell M. S. Meck W. H. (2004). Cortico-striatal circuits and interval timing: coincidence detection of oscillatory processes. *Brain Res. Cogn. Brain Res.* 21 139–170. 10.1016/j.cogbrainres.2004.06.012 15464348

[B70] Merchant H. Zarco W. Pérez O. Prado L. Bartolo R. (2011). Measuring time with different neural chronometers during a synchronization-continuation task. *Proc. Natl. Acad. Sci. U.S.A.* 108 19784–19789. 10.1073/pnas.1112933108 22106292 PMC3241773

[B71] Milo R. Shen-Orr S. Itzkovitz S. Kashtan N. Chklovskii D. Alon U. (2002). Network motifs: simple building blocks of complex networks. *Science* 298 824–827. 10.1126/science.298.5594.824 12399590

[B72] Newman M. E. J. (2006). Finding community structure in networks using the eigenvectors of matrices. *Phys. Rev. E* 74:036104. 17025705 10.1103/PhysRevE.74.036104

[B73] O’Connor D. Potler N. V. Kovacs M. Xu T. Ai L. Pellman J. (2017). The healthy brain network serial scanning initiative: a resource for evaluating inter-individual differences and their reliabilities across scan conditions and sessions. *Gigascience* 6 1–14. 10.1093/gigascience/giw011 28369458 PMC5466711

[B74] Onslow A. C. E. Bogacz R. Jones M. W. (2011). Quantifying phase-amplitude coupling in neuronal network oscillations. *Prog. Biophys. Mol. Biol.* 105 49–57. 10.1016/j.pbiomolbio.2010.09.007 20869387

[B75] Patel A. D. (2010). *Music, Language, and the Brain.* Oxford: Oxford University Press.

[B76] Pinker S. (1997). *How the Mind Works.* New York, NY: Norton. 10.1111/j.1749-6632.1999.tb08538.x 10415890

[B77] Poldrack R. A. Baker C. I. Durnez J. Gorgolewski K. J. Matthews P. M. Munafò M. R. (2017). Scanning the horizon: towards transparent and reproducible neuroimaging research. *Nat. Rev. Neurosci.* 18 115–126. 10.1038/nrn.2016.167 28053326 PMC6910649

[B78] Reichardt J. Bornholdt S. (2006). Statistical mechanics of community detection. *Phys. Rev. E* 74:016110. 10.1103/PhysRevE.74.016110 16907154

[B79] Rubinov M. Sporns O. (2010). Complex network measures of brain connectivity: uses and interpretations. *NeuroImage* 52 1059–1069. 10.1016/j.neuroimage.2009.10.003 19819337

[B80] Salimi-Khorshidi G. Douaud G. Beckmann C. F. Glasser M. F. Griffanti L. Smith S. M. (2014). Automatic denoising of functional MRI data: combining independent component analysis and hierarchical fusion of classifiers. *NeuroImage* 90 449–468. 10.1016/j.neuroimage.2013.11.046 24389422 PMC4019210

[B81] Schack B. (2004). How to construct a microstate-based alphabet for evaluating information processing in time. *Int. J. Bifurc. Chaos* 14 793–814. 10.1142/s0218127404009478

[B82] Sethares W. A. (1998). *Tuning, Timbre, Spectrum, Scale.* London: Springer.

[B83] Sethares W. A. (2007). *Rhythm and Transforms.* London: Springer-Verlag.

[B84] Shirazi A. H. Jafari G. R. Davoudi J. Peinke J. Tabar M. R. R. Sahimi M. (2009). Mapping stochastic processes onto complex networks. *J. Stat. Mech. Theory Exp.* 2009:07046.

[B85] Smith S. M. Andersson J. Auerbach E. J. Beckmann C. F. Bijsterbosch J. Douaud G. (2013). Resting-state fMRI in the human connectome project. *NeuroImage* 80 144–168. 10.1016/j.neuroimage.2013.05.039 23702415 PMC3720828

[B86] Smith S. M. Miller K. L. Salimi-Khorshidi G. Webster M. Beckmann C. F. Nichols T. E. (2011). Network modelling methods for fMRI. *NeuroImage* 54 875–891. 10.1016/j.neuroimage.2010.08.063 20817103

[B87] Smith S. M. Nichols T. E. (2018). Statistical challenges in “Big Data” human neuroimaging. *Neuron* 97 263–268. 10.1177/1740774509105380 29346749

[B88] Sporns O. (2011a). The human connectome: a complex network. *Ann. N. Y. Acad. Sci.* 1224 109–125. 10.1111/j.1749-6632.2010.05888.x 21251014

[B89] Sporns O. (2011b). The non-random brain: efficiency, economy, and complex dynamics. *Front. Comput. Neurosci.* 5:5. 10.3389/fncom.2011.00005 21369354 PMC3037776

[B90] Sporns O. (2012). From simple graphs to the connectome: networks in neuroimaging. *NeuroImage* 62 881–886. 10.1016/j.neuroimage.2011.08.085 21964480

[B91] Sporns O. Tononi G. Kötter R. (2005). The human connectome: a structural description of the human brain. *PLoS Comput. Biol.* 1:e42. 10.1371/journal.pcbi.0010042 16201007 PMC1239902

[B92] Stanley M. L. Moussa M. N. Paolini B. Lyday R. G. Burdette J. H. Laurienti P. J. (2013). Defining nodes in complex brain networks. *Front. Comput. Neurosci.* 7:169. 10.3389/fncom.2013.00169 24319426 PMC3837224

[B93] Termenon M. Jaillard A. Delon-Martin C. Achard S. (2016). Reliability of graph analysis of resting state fMRI using test-retest dataset from the Human Connectome Project. *NeuroImage* 142 172–187. 10.1016/j.neuroimage.2016.05.062 27282475

[B94] Van Essen D. C. Smith S. M. Barch D. M. Behrens T. E. J. Yacoub E. Ugurbil K. (2013). The WU-Minn human connectome project: an overview. *NeuroImage* 80 62–79. 10.1016/j.neuroimage.2013.05.041 23684880 PMC3724347

[B95] Varela F. Lachaux J. P. Rodriguez E. Martinerie J. (2001). The brainweb: phase synchronization and large-scale integration. *Nat. Rev. Neurosci.* 2 229–239. 10.1038/35067550 11283746

[B96] Varoquaux G. Craddock R. C. (2013). Learning and comparing functional connectomes across subjects. *NeuroImage* 80 405–415. 10.1016/j.neuroimage.2013.04.007 23583357

[B97] Ville D. V. D. Britz J. Michel C. M. (2010). EEG microstate sequences in healthy humans at rest reveal scale-free dynamics. *Proc. Natl. Acad. Sci. U.S.A.* 107 18179–18184. 10.1073/pnas.1007841107 20921381 PMC2964192

[B98] Voss H. U. Timmer J. Kurths J. (2004). Nonlinear dynamical system identification from uncertain and indirect measurements. *Int. J. Bifurc. Chaos* 14 1905–1933. 10.1142/s0218127404010345

[B99] Wiener M. Kanai R. (2016). Frequency tuning for temporal perception and prediction. *Curr. Opin. Behav. Sci.* 8 1–6. 10.1016/j.cobeha.2016.01.001

[B100] Zhang H. Watrous A. J. Patel A. Jacobs J. (2018). Theta and alpha oscillations are traveling waves in the human neocortex. *Neuron* 98 1269–1281. 10.1016/j.neuron.2018.05.019 29887341 PMC6534129

[B101] Zuo X.-N. Biswal B. B. Poldrack R. A. (2019a). Editorial: reliability and reproducibility in functional connectomics. *Front. Neurosci.* 13:117. 10.3389/fnins.2019.00117 30842722 PMC6391345

[B102] Zuo X.-N. Xu T. Milham M. P. (2019b). Harnessing reliability for neuroscience research. *Nat. Hum. Behav.* 3 768–771. 10.1038/s41562-019-0655-x 31253883

[B103] Zuo X.-N. Di Martino A. Kelly C. Shehzad Z. E. Gee D. G. Klein D. F. (2010). The oscillating brain: complex and reliable. *NeuroImage* 49 1432–1445. 10.1016/j.neuroimage.2009.09.037 19782143 PMC2856476

[B104] Zuo X.-N. Xing X.-X. (2014). Test-retest reliabilities of resting-state FMRI measurements in human brain functional connectomics: a systems neuroscience perspective. *Neurosci. Biobehav. Rev.* 45 100–118. 10.1016/j.neubiorev.2014.05.009 24875392

